# Iraqi Kurdish EFL teachers’ beliefs about technological pedagogical and content knowledge: The role of teacher experience and education

**DOI:** 10.3389/fpsyg.2022.969195

**Published:** 2022-10-18

**Authors:** Sirwan Sadiq Ali, Behbood Mohammadzadeh

**Affiliations:** ELT Department, Faculty of Education, Cyprus International University, Nicosia, Turkey

**Keywords:** EFL teachers, teachers’ beliefs, experienced teachers, novice teachers, TPACK

## Abstract

Exploring technological pedagogical and content knowledge (TPACK) has obtained considerable importance over the past 2 years when education needs to rely on using online learning platforms due to the COVID pandemic. Teachers’ beliefs could play a determining role in their decisions and the ways they implement their knowledge. It has, however, been indicated that teachers’ beliefs about TPACK in language pedagogy merits additional empirical evidence, especially through a mixed-methods design. To this aim, this study probed teachers’ beliefs towards TPACK in general and its components in particular in Iraqi Kurdistan. Additionally, the role of teaching experience and education degree in affecting the teachers’ beliefs was explored. The data was collected through a questionnaire responded by 105 EFL (English a Foreign Language) teachers and enriched by qualitative data gathered through a structured interview. Overall, it was found that teachers generally had a higher level of pedagogy and subject matter knowledge than technological knowledge. Although the quantitative data indicated that the experienced teachers had significantly higher pedagogical, content, technological, and pedagogical content knowledge than the novice teachers, the qualitative analysis revealed that novice teachers were more skilled in the utilization of technology-related knowledge domains. Furthermore, Ph.D. participants demonstrated higher level of TPACK confidence than the BA ones. The findings are discussed in the light of theories on teachers’ beliefs, and implications for teachers and teacher educators are presented.

## Introduction

Technology has permeated every aspect of our lives and become an integral part of it. As learners in the digital-native generation think differently from those of the former century (Prensky, 2001; Bennett et al., 2008), demands for implementing technology have increased dramatically (Chauhan, 2017; Yenkimaleki and van Heuven, 2019; Raygan and Moradkhani, 2020). This paradigm shift in learning and the ongoing Coronavirus Disease 2019 (COVID-19) pandemic have led to a dramatic rise in the demand for rethinking traditional pedagogical practices to provide responsive, relevant, and effective learning (Cahapay, 2020; Adipat, 2021). In response to this need, researchers have been prominently concerned with how language teachers deliver language learning materials in technology-enhanced classrooms following the principles of effective pedagogy (Tseng et al., 2020; Moser and Wei, 2021). In such a context, teachers’ pedagogical beliefs about integrating technology as an emerging theme in explicating teachers’ pedagogical reasoning and employing digital technology have been of primary importance (Jimoyiannis and Komis, 2007; Tseng et al., 2020).

Technological pedagogical and content knowledge (TPACK) is a comprehensive framework that identifies well-defined types of knowledge required for effective teaching of subject matter knowledge enhanced by technology adaptation (Mishra and Koehler, 2006). TPACK has emerged as a critical notion to determine knowledge and skills teachers need to promote learning and maximize knowledge and skill acquisition (Schmidt et al., 2009; West et al., 2017; Habibi et al., 2019; Raygan and Moradkhani, 2020; Santos and Castro, 2021). Although TPACK framework was proposed as a robust framework, studies on TPACK show that it has not transformed technology integration in teaching yet, which could be affected by teachers’ beliefs (Heitink et al., 2017; Chai et al., 2019). Thus, exploring teachers’ beliefs about TPACK could help us develop a deeper understanding into issues surrounding technology integration in language teaching and learning (e.g., Chai et al., 2019; Jin et al., 2021).

According to Ellis (2012), “any understanding of how teachers teach requires an examination of their beliefs about teaching” (p. 146). Thus, being an inseparable part of teaching practices, teachers’ beliefs largely shape how teachers are engaged with classroom activities and incorporate strategies to respond to ‘problems of practice’ (Skott, 2014, p. 19). Given this importance, it seems that identifying teachers’ beliefs about TPACK could help resolve potential ambiguities frequently occurring in teachers’ pedagogical practices. Recent research on technology and teacher self-efficacy (Teo et al., 2018), teachers’ design beliefs (Chai et al., 2019), beliefs of notable users of technology (Ertmer et al., 2012), pedagogy in different subjects (Szeto and Cheng, 2017), technology-based reading (Gunbas and Gozukucuk, 2020), and preparation practices (Voithofer and Nelson, 2021) shows a strong link between technology integration and teachers pedagogical beliefs. Although teachers’ beliefs influence various areas in language teaching, to date, no study has examined teachers’ beliefs towards TPACK. Therefore, the present study aims at examining teachers’ pedagogical beliefs not only about technology integration but also about content and pedagogical knowledge and the intersection among these. It also investigates the effects of teaching experience and academic degrees on different aspects of TPACK from quantitative and qualitative perspectives.

## Literature review

### TPACK framework

Over the past few decades, teaching with technology has dramatically changed. This is because integrating technology with learning could pave the way for providing learners with more effective learning (Ponce Gea et al., 2021); more recently, employing technology has become an important goal of educational organizations in order to ensure quality education during the pandemic (Tria, 2020; Adipat, 2021). Since the beginning of the COVID-19 pandemic, there has been an abrupt shift towards distance-learning; however, teachers find technology-dependent teaching a daunting task due to their lack of experience in teaching remote classes (Moser et al., 2021; Moser and Wei, 2021). This dramatic shift poses an increasing demand for rethinking the contribution of technology to teaching, which specifically requires developing a stronger grasp of subject matter knowledge (Cahapay, 2020; Adipat, 2021). To investigate teachers’ contribution to this significant shift, understanding teachers beliefs about and integration of information and communication technologies (ICT) is a critical step to take.

To examine teachers’ knowledge of teaching, Shulman (1986) proposed the concept of pedagogical content knowledge (PCK). PCK is “an understanding of how particular topics, problems, or issues are organized, presented, and adapted to the diverse interests and abilities of learners, and presented for instruction” (p. 8). According to the model, teachers’ effectiveness is not merely contingent on the content knowledge (CK); it should also take pedagogical knowledge (PK) into account. He stressed that pedagogy and CK are combined for the purpose of “representing and formulating the subject that make it comprehensible to others” (p. 9). With the advent of digital technologies, Mishra and Koehler (2006) added technology as a third pillar to the PCK framework in order to form TPACK. They stated that the TPACK had been proposed as “a conceptual framework for educational technology by building on Shulman’s formulation of ‘PCK’ and extend it to the phenomenon of teachers integrating technology into their pedagogy” (p. 1017). TK is a general and learning oriented cognizance of computer and mobile software and hardware, awareness and employment of specific technologies, and solving technical problems (Adipat, 2021). Thus, by adding Technological knowledge (TK) to the framework, new sub-components have stemmed from the framework, including overlapping elements of TK, PK, and CK (Mishra and Koehler, 2006; Koehler et al., 2007). The first sub-domain is PCK which represents teachers’ knowledge of subject matter knowledge and strategies required for teaching the intended subject matter. Secondly, Technological Content Knowledge (TCK) is the affordance of subject-specific technology embodied in the “choice of technologies affords and constrains the types of content ideas that can be taught” (Mishra and Koehler, 2008, p. 7). Technological pedagogical knowledge (TPK) is the third element representing the knowledge of understanding and using various technologies in the act of teaching through the application of appropriate technologies aimed at bringing improvements to pedagogical settings. The last sub-component is TPACK explicating an interplay between the main components of CK, PK, and TK. It insists on “discovering and describing how technology-related professional knowledge is implemented and instantiated in practice” (Koehler and Mishra, 2009, p. 67).

It is important to note that ICT is more integrally related to TK and TPK but loosely related to CK and PCK (Chen and Jang, 2019). In addition to aforementioned knowledge (i.e., TK, TPK, CK, and PCK), TPACK encompasses PK, TCK, and TPACK. For this reason, TPACK framework has been widely accepted as a conceptual framework by teachers and researchers (e.g., Graham, 2011; Chai et al., 2016; Koh and Chai, 2016; Tseng et al., 2020). According to Aniq and Drajati (2019), successful technology integration relies heavily on teachers’ TPACK. This is because the framework represents a complex interplay of particularly vital knowledge areas (i.e., content, pedagogy, and technology) and may accurately predict teachers’ intentions of implementing technology (Hsu, 2016; Hsu et al., 2021). According to Mishra and Koehler (2006), the seven-factor model is the most widely cited framework, and recent research (e.g., Chai et al., 2016; Harris et al., 2017) reassured the identification of the seven factors with enough validity and reliability in the light of evidence.

Research on TPACK framework adopted different measures for investigating distinct areas of knowledge among preservice (e.g., Koh et al., 2010; Chai, et al., 2019; Singh and Kasim, 2019; Gunbas and Gozukucuk, 2020) and in-service teachers (e.g., Archambault and Barnett, 2010; Lee and Tsai, 2010; Ertmer et al., 2012; Anderson and Putman, 2020), or both (e.g., Dong et al., 2015; Nazari et al., 2019). In addition, studies have been conducted in various countries including China (e.g., Dong et al., 2015), Taiwan (e.g., Jang and Chang, 2016), the USA (e.g., Lee and Tsai, 2010; Ertmer et al., 2012), Singapore (e.g., Koh et al., 2010), and Iran (e.g., Nazari et al., 2019). Although the number of TPACK studies has increased from 2011 onwards (Tseng et al., 2020), there is a dearth of research in Iraqi Kurdistan addressing preservice and in-service teachers TPACK knowledge (Koh et al., 2010; Tseng et al., 2020).

### Teachers’ beliefs and TPACK

Teachers’ belief is a crucial factor influencing teachers’ actions and decision-making; teachers’ choices and decisions about *whats* and *hows* of teaching rely heavily on a network of their beliefs, thoughts, and knowledge (Borg, 2003; Chai et al., 2019). In other words, teachers’ beliefs can greatly influence what teachers do and how learning opportunities for learners are either created or curbed (Burns et al., 2015).

Concerning TPACK, research has drawn attention to the relevant importance between teachers’ beliefs and technology integration (e.g., Jimoyiannis and Komis, 2007; Dong et al., 2015; Paratore et al., 2016; Gil-Flores et al., 2017; Tondeur et al., 2017; Vereshchahina et al., 2018; Chai et al., 2019; Anderson and Putman, 2020; Gunbas and Gozukucuk, 2020). However, recent reviews of TPACK studies indicate the dearth of research on the relationship between teachers’ beliefs and TPACK knowledge (Voogt et al., 2013; Wu, 2013; Willermark, 2018). These review studies also indicate that very few studies have examined domain-specific TPACK targeting language teaching.

Researchers have been keen to understand the role that teachers’ beliefs play in exploiting modern technology in the classroom. For instance, Ertmer et al. (2012) conducted a study on a small number of teachers recognized as notable users of technology. They found that teachers’ beliefs and interests greatly impact implementing technology. In another study, Luik et al. (2018) examined preservice teachers’ perceptions of the three knowledge bases of TPACK (i.e., content, pedagogy, and technology), and found that although the teachers were good at integrating technology, they lacked PK. The study also illustrated that MA students generally had higher level of perceptions on the knowledge bases than the BA level participants.

However, to date, no studies have investigated teachers’ professional experience, academic degrees, and beliefs in relation to TPACK using a mixed methods design. Therefore, to shed lights on how teachers’ beliefs contribute to TPACK, the present study addressed the following questions:

1. What are Iraqi Kurdish EFL teachers’ general beliefs, understandings, and contextual uses of TPACK?
2. Do experienced and novice teachers’ beliefs towards TPACK and their uses of it significantly differ?
3. Does teachers’ academic degree significantly affect the teachers’ beliefs towards TPACK?

## Materials and methods

### Design

Explanatory Sequential Mixed Methods Design was adopted in the present study. Quantitative and qualitative data were collected to address the research questions; the quantitative data was collected through a questionnaire and qualitative data was collected through structured interviews.

### Setting

In the Kurdistan Region of Iraq (KRI), English is considered an essential subject in the educational system and a medium of instruction in a number of fields of study. English is also intensively taught in the private institutes to raise learners’ communicative competence and to ensure success in future academic and vocational experience. As many Kurds consider English a key factor to educational and economic growth, there is an increasing demand for a high quality English language teaching in the KRI. During the pandemic attempts have been made by governmental and private institutions to develop quality teaching in the region. As a result, Kurdish EFL teachers are expected to have a better grasp of TPACK in their teaching practice.

### Participants

A total of 105 EFL teachers (Male = 71, Female = 34) were recruited through a non-probability sampling method of convenience and snowball sampling in order to participate in the present study. These methods, convenience and snowball sampling, are employed to reach a desired number of participant through selecting individuals who fit the purpose of the study (Emerson, 2015). The participants had different academic degrees teaching English in schools, institutes and universities in Erbil and Sulaimani, Iraq. Their age ranged between 23 and 50 years old; they held different academic degrees, including BA (*N* = 29), MA (*N* = 56), and Ph.D. (*N* = 20). The participants were categorized as novice and experienced teachers. According to Gatbonton’s criteria (Gatbonton, 2008), teachers with less than 2 years of experience were regarded as novice whilst those with at least 4 years of teaching experience were considered experienced. Based on the criteria, 77 teachers were regarded as experienced and 28 as novice teachers. For the qualitative phase, the teachers were selected based on their years of experience in language pedagogy. To this end, 10 teachers (5 Experienced, 5 Novice) who were MA holders in English language were recruited.

### Instruments

The TPACK questionnaire developed by Baser et al.’s (2016) was used to collect the data. The questionnaire includes 39 items (TK had 9 items, CK 5 items, PK 6 items, PCK 5 items, TCK 3 items, TPK 7 items, and TPACK 4 items) of nine point Likert-scale ranging from nothing (1) to a great deal (9). Internal consistency of the TPACK questionnaire, measured through Cronbach’s alpha, was reported to be 0.92, which is high.

To look into the issue from a qualitative perspective, a structured face-to-face interview was conducted with 10 experienced and novice teachers. The interviews were continued until data saturation happened. An interview checklist was used to make the interviews more systematic, yet whenever the teachers’ responses were terse, the interviewer asked follow-up questions to elicit more detailed answers.

### Data collection procedures

After obtaining the consent of the participants, they were informed about the general purpose of the study. Afterwards, they were given the questionnaire directly in face-to-face meetings or through email. Having collected the questionnaires, a follow-up, face-to-face interview was carried out to explore teachers’ response to the questionnaire in more detail. The interview was conducted with 10 of the teachers who were willing to attend the interview. The interviewees were guaranteed of complete anonymity; they were informed that data would remain confidential and would be used anonymously. The interviews were audio recorded and later transcribed verbatim; they lasted 23 min on average.

## Results

The first research question addressed the participating teachers’ beliefs about TPACK. The results of the questionnaire highlighted that the teachers had a higher level of CK and PCK with the means of 8.14 and 7.81, respectively. In contrast, the lowest mean was obtained for TPACK with 6.50 and TK with 6.72 (Table 1). In terms of teachers’ beliefs regarding items with the highest and lowest mean level, it was found that subcomponents of CK category, i.e., item 13 (*ˉx* = 8.32), item 10 (*ˉx* = 8.20), and item 12 (*ˉx* = 8.15) had the highest means, respectively. Conversely, the first two lowest items belong to TK and the third one is related to TPACK; items with lowest means were item 8 (*ˉx* = 4.88), item 7 (*ˉx* = 5.52), and item 36 (*ˉx* = 6.10).

**Table 1:** Descriptive statistics for teachers’ overall beliefs towards TPACK.

TPACK scales	Minimum	Maximum	Mean	SD
Content knowledge (CK)	3.20	9.00	8.1448	1.22459
Pedagogical content knowledge (PCK)	3.00	9.00	7.8114	1.13471
Pedagogical knowledge (PK)	3.67	9.00	7.5841	1.21558
Technological pedagogical knowledge (TPK)	2.86	9.00	7.2939	1.29754
Technological content knowledge (TCK)	2.67	9.00	7.1714	1.41883
Technological knowledge (TK)	2.56	9.00	6.7206	1.60906
Technological pedagogical and content knowledge (TPACK)	2.75	9.00	6.5095	1.57654

To find more about what teachers’ notions of TPACK are associated with, interviews were carried out. Firstly, it was found that most of the teachers provided obvious clues about PK; they generally know and understand the meaning of PK. However, it should be noted that one third of the teachers could not draw a correct representation of PK; they considered PK as the subject matter knowledge, a merely communicative approach in language teaching, or linguistic knowledge; all of which are related to CK (Table 2).

**Table 2:** Experienced and novice teachers’ beliefs about TPACK components.

TPACK	Experienced teachers	Novice teachers
PK	**For experienced teachers this component implied:**	**For novice teachers this component implied:**
	Discussing subjects among students	Having linguistic knowledge
	Guiding and explaining complicated concepts	Adopting communicative approach
	Knowing how to teach	Being a way of teaching language skills
	Practicing Communicative language learning	Using appropriate ways of teaching
CK	Having common knowledge about English	Having general teaching skills
	Knowing about context	Knowing about culture
	Having cultural knowledge	Mastering the four language skills
	Being the four skills, grammar, semantics and pragmatics	Being a good speaker
TK	Making classroom more enjoyable	Mastering new technologies
	Using mostly traditional apps and online platforms	Being very helpful in teaching and for entertainment
	Listening to native speakers	Motivating students and saving time
	Motivating students	Teaching without it will be very difficult
	Being an effective and faster way to teach	Making learners learn faster and better
		Practicing listening to native speakers
		Keeping you in contact with students
		Using both learning oriented apps and online platforms
TPK	Teaching them to use dictionaries and websites	Giving assignments like watching videos for class discussion
	Encouraging students to use technology	Encouraging them to listen to various materials in English
	Using emails and Messenger groups	Assigning activities to be done online
		Instructing them to use educational websites
TCK	Teaching them to search online	Using educational apps for distance learning
	Making contact with students while being at home	Sharing questions through online groups
	Sharing videos and e-books	Encouraging them while they are at home
	Introducing applications and videos	Communicating *via* online platforms
PCK	Teaching effectively without technology is impossible	Teaching without technology is impossible
	Teaching without technology is boring	Ignoring technology is meaningless
TPACK	Using and integrating technology in teaching	Integrating technology with content and pedagogy
	Being a beneficial framework	Following this framework is extremely important
	Implementing technology to do some activities	Using online and offline facilities to do most of the activities

Secondly, we found that teachers had a clear conception of what was possible to be linked with CK; nearly all of them tied CK to a variety of language skills and subskills, including the four primary language skills and the subskills like pronunciation, vocabulary, conversation, and grammar. The aforementioned skills and subskills are all components of CK. Nevertheless, some of the respondents commented that CK is knowledge about culture, context, native speakers’ context, and background knowledge of the teacher.

Thirdly, teachers expressed strong positive views about TK and its contribution to language pedagogy; they stated that they had frequently used technological tools in their teaching practice on a daily basis. The interviewees provided various reasons to demonstrate the impact of integrating technology in language teaching such as facilitating learning and motivating students. Regarding their pedagogical practices, the teachers expressed their genuine willingness to employ technology in carrying out classroom activities; they added that technology plays a crucial role in “making classrooms a more enjoyable learning environment,” and it is “very helpful in teaching and entertaining students.”

### Experienced versus novice teachers’ TPACK

The second question examined novice and experienced teachers’ beliefs about TPACK. The descriptive statistics and *t*-test results for comparison of experienced and novice teachers’ beliefs about TPACK are presented in Table 3. The analysis of the total scores demonstrated that there existed a statistically significant difference between the performance of experienced and novice participants, *p* < 0.05. It could be noticed in Table 3 that there are significant differences between experienced and novice teachers with regards to TK, CK, PK, and PCK. The results in Table 3 suggest that no significant difference exists between experienced and novice teachers for TCK, TPK, and TPACK. As to which group of teachers had higher scores across TPACK components, independent sample T-test was administered. It was observed that experienced teachers generally have higher score across all the scales; it was found that experienced teachers have significantly higher CK, PK, TK, and PCK (Table 4). However, the difference between experienced and novice teachers for TPK, TCK, and TPACK did not reach significance.

**Table 3:** Independent samples test comparing novice and experienced teachers’ TPACK.

	Levene’s test for equality of variances		*t*-Test for equality of means						
	*F*	Sig.	*T*	df	Sig. (2-tailed)	Mean difference	Std. error difference	95% Confidence interval of the difference	
								Lower	Upper
TPACK	0.160	0.690	0.351	103	0.726	0.12256	0.34939	−0.57037	0.81550
			0.362	50.961	0.719	0.12256	0.33864	−0.55730	0.80243
TK	0.143	0.706	2.709	103	0.008	0.93398	0.34474	0.25027	1.61769
			2.788	50.692	0.007	0.93398	0.33502	0.26131	1.60665
PK	1.332	0.251	2.451	103	0.016	0.64232	0.26202	0.12266	1.16198
			2.345	44.270	0.024	0.64232	0.27395	0.09030	1.19434
CK	2.386	0.125	2.562	103	0.012	0.67468	0.26329	0.15250	1.19685
			2.211	37.829	0.033	0.67468	0.30515	0.05684	1.29251
PCK	0.004	0.948	2.161	103	0.033	0.53182	0.24611	0.04372	1.01991
			2.162	47.982	0.036	0.53182	0.24603	0.03713	1.02650
TCK	0.485	0.488	1.163	103	0.247	0.36364	0.31258	−0.25629	0.98357
			1.283	58.788	0.205	0.36364	0.28342	−0.20353	0.93080
TPK	1.490	0.225	1.656	103	0.101	0.47032	0.28398	−0.09288	1.03351
			1.811	57.635	0.075	0.47032	0.25975	−0.04971	0.99034

**Table 4:** Mean difference of experienced and novice teachers’ TPACK.

	Experience	*N*	Mean	Std. deviation	Std. error mean
TPACK	Experienced	77	6.5422	1.60970	0.18344
	Novice	28	6.4196	1.50624	0.28465
TK	Experienced	77	6.9697	1.58613	0.18076
	Novice	28	6.0357	1.49257	0.28207
PK	Experienced	77	7.7554	1.15615	0.13176
	Novice	28	7.1131	1.27096	0.24019
CK	Experienced	77	8.3247	1.07314	0.12230
	Novice	28	7.6500	1.47936	0.27957
PCK	Experienced	77	7.9532	1.11539	0.12711
	Novice	28	7.4214	1.11467	0.21065
TCK	Experienced	77	7.2684	1.48485	0.16921
	Novice	28	6.9048	1.20307	0.22736
TPK	Experienced	77	7.4193	1.34399	0.15316
	Novice	28	6.9490	1.11012	0.20979

In order to probe into novice and experienced teachers’ beliefs about TPACK in more depth, 10 teachers (five experienced and five novice teachers) were interviewed until data saturation happened. The interviewees were MA holders of English. They were interviewed and the data was then transcribed and coded. Table 2 presents novice and experienced teachers’ reflection on each component of TPACK. As shown in Table 2, further insights into teachers’ view on TPACK emerged from the interviews. The interviews implied that the experienced and novice teachers had some misconceptions about the PK. The experienced teachers stated that PK is ‘subject matter knowledge’, ‘guiding and explaining complicated concepts’, and ‘practicing communicative language learning.’ In contrast, novice teachers added ‘linguistic knowledge’, ‘adopting communicative approach’, and ‘being a way of teaching language skills’ while describing PK. Although some of the responses reflected that PK is engagement with pedagogical practices, none of them offered a satisfactory definition encompassing pedagogy in language teaching.

Concerning the experienced teachers’ reflection on CK, they stated that it is general ‘language knowledge’, ‘the four main skills’ and subskills (i.e., grammar and vocabulary), ‘semantics and pragmatics,’ as well as knowledge about culture. The majority of the aforementioned skills and subskills are considered as accurate representations of CK. However, some major misconceptions were found in novice teachers’ understanding of CK. For instance, knowledge about culture and being a good speaker was considered by some teachers as evidence of CK while, in fact, they are not significant indicators of CK.

As summarized in Table 2, teacher’s responses show that teachers were more positive about TK and indicated a thorough understanding of this concept. Both groups of teachers provided the basis for integrating technology stating that it facilitates learning, saves time, and motivates learners. Having approved the efficient use of technology, novice teachers demonstrated their interest and enthusiasm to make the full use of it. With respect to types of technology they used in their teaching, the participants referred to a wide range of technologies including Messenger groups, Viber, YouTube, Telegram, email, Facebook, Google, blogs, projector, speaker, iPad, Padlet, e-books, laptop, Zoom Meeting, Kahoot, PowerPoint, Edmodo, smart board, and offline dictionaries. Notably, few teachers referred to their use of educationally designed programs like Zoom Meeting, Kahoot, and Edmodo.

Analysis of TPACK subdomains (i.e., TPK, TCK, and PCK) highlighted that teachers were positively disposed to these concepts. Firstly, to discover how teachers combine technology and pedagogy as a mean of teaching (i.e., TPK), the experienced teachers stated that they provide learners with guidelines, and recommend employing technology; some of them even named the tools and devices to be used. In contrast, the novice teachers provided more specific examples; they give assignments, instructions, and actively encouraged their students to employ technology in language learning. Secondly, to address integrating subject matter knowledge with technology (i.e., TCK), the majority of the participants had positive beliefs towards TCK and provided obvious examples of how they associate technology with CK; however, novice teachers were more bound to the use of online platforms such as Messenger, email, and Zoom Meeting. Thirdly, probing teachers’ beliefs about teaching subject matter knowledge without using technology (i.e., PCK), teachers either felt at ease to ignore technology, or they ‘could not’ even teach without it. Overall, both groups thought (at least to some extent) that they consider these types of knowledge in their language teaching practices and provided a range of examples to further evidence that they viewed the TPACK subdomains as skills and abilities needed in language pedagogy.

### TPACK knowledge of BA, MA, and Ph.D. teachers

To find the impact of education on TPACK, teachers’ academic degrees were observed. The responses of participating teachers (BA, *N* = 29, MA, *N* = 56, Ph.D., *N* = 20) were compared to find the differences between categorical independent variables on the continuous dependent variable using analysis of variance (ANOVA). Table 5 presents descriptive analysis of teachers’ beliefs about TPACK to each scale. To examine whether there is a significant difference among BA, MA and Ph.D. participants, a one-way analysis of ANOVA was run (see Table 6). The analysis suggests that the three groups differed significantly in terms of their total TPACK scores *p* < 0.05. One-way analysis of different components of TPACK, however, demonstrated that the three groups only differed in terms of TK *p* < 0.05. As for the other components, no significant difference was observed.

**Table 5:** Descriptive statistics for BA,MA, and PhD participant’s TPACK.

		*N*	Mean	SD	Std. error	95% Confidence interval for mean		Min	Max
						Lower bound	Upper bound		
TPACK	Ph.D.	20	6.9750	1.41863	0.31721	6.3111	7.6389	3.00	8.75
	MA	56	6.4955	1.60148	0.21401	6.0667	6.9244	2.75	9.00
	BA	29	6.2155	1.60735	0.29848	5.6041	6.8269	2.75	9.00
TK	Ph.D.	20	7.4611	1.36048	0.30421	6.8244	8.0978	4.22	9.00
	MA	56	6.9028	1.42875	0.19092	6.5202	7.2854	3.00	9.00
	BA	29	5.8582	1.76706	0.32813	5.1861	6.5304	2.56	8.33
PK	Ph.D.	20	7.9417	0.84514	0.18898	7.5461	8.3372	6.17	9.00
	MA	56	7.5000	1.37950	0.18434	7.1306	7.8694	3.67	9.00
	BA	29	7.5000	1.07367	0.19938	7.0916	7.9084	5.00	9.00
CK	Ph.D.	20	8.4900	0.82200	0.18380	8.1053	8.8747	6.20	9.00
	MA	56	8.1286	1.31864	0.17621	7.7754	8.4817	3.20	9.00
	BA	29	7.9379	1.25140	0.23238	7.4619	8.4139	4.20	9.00
PCK	Ph.D.	20	8.2500	0.86298	0.19297	7.8461	8.6539	6.20	9.00
	MA	56	7.7857	1.18957	0.15896	7.4671	8.1043	3.00	9.00
	BA	29	7.5586	1.13689	0.21111	7.1262	7.9911	3.80	9.00
TCK	Ph.D.	20	7.7833	1.01610	0.22721	7.3078	8.2589	5.67	9.00
	MA	56	7.0417	1.50294	0.20084	6.6392	7.4442	3.00	9.00
	BA	29	7.0000	1.41702	0.26313	6.4610	7.5390	2.67	9.00
TPK	Ph.D.	20	7.7000	0.95540	0.21363	7.2529	8.1471	5.43	9.00
	MA	56	7.2806	1.39279	0.18612	6.9076	7.6536	2.86	9.00
	BA	29	7.0394	1.27997	0.23769	6.5525	7.5263	3.86	8.86

**Table 6:** ANOVA examining the differences among BA, MA and Ph.D. participants regarding TPACK.

		Sum of squares	df	Mean square	*F*	Sig.
TK	Between Groups	34.392	2	17.196	7.468	0.001
	Within Groups	234.870	102	2.303		
Average CK	Between Groups	1.123	2	0.562	1.218	0.300
	Within Groups	47.013	102	0.461		
Average PK	Between Groups	1.404	2	0.702	1.070	0.347
	Within Groups	66.896	102	0.656		
Average PCK	Between Groups	1.771	2	0.885	2.283	0.107
	Within Groups	39.558	102	0.388		
Average TCK	Between Groups	1.032	2	0.516	2.366	0.099
	Within Groups	22.231	102	0.218		
Average TPK	Between Groups	3.137	2	1.569	1.557	0.216
	Within Groups	102.784	102	1.008		
Average TPACK	Between Groups	1.353	2	0.677	1.389	0.254
	Within Groups	49.707	102	0.487		
Average total score = mean (Item 1 to Item 39)	Between Groups	8.644	2	4.322	3.410	0.037
	Within Groups	129.271	102	1.267		

To pinpoint which groups differed from each other, Tukey *post hoc* test was conducted. *Post hoc* analysis of participants’ total TPACK scores showed that Ph.D. participants have significantly higher score in the average total score than BA participants with the mean difference of 0.85. The analysis also showed significant differences among the groups in terms of TK. The Ph.D. and MA participants had a higher level of TK than BA degree participants with the mean difference of 1.60 and 1.04, respectively. Concerning MA and Ph.D. participants, no significant difference was observed.

## Discussion

Regarding teachers’ general beliefs and understanding of TPACK, the results of the quantitative phase indicated that the highest score was obtained for CK and PCK, and the lowest score was for TPK and TK. The participants not only rated CK high in the questionnaire but also found it easy to identify in the interview. This could be due to familiarity with the concept and having casual encounters with various subject matters in their teaching on the daily basis. This accords with the findings of Chen and Jang (2019) who found that teachers obtained the highest score for CK and PCK.

Concerning the meaning of PK, the teachers could define different types of approaches and techniques such as communicative approach, teaching procedures, and providing explanations; all of these reflections are directly related to PK. However, it should be noted that sometimes they referred to elements of the subject matter knowledge while they were describing PK. Overall, they were unable to express a clear definition of PK, and some of them considered PK as a certain skill, strategy, or a teaching method, not an umbrella term covering knowledge transition in general. For example, when they were asked about the meaning of PK, one of the teachers stated the following:

In my opinion, the teaching process firstly starts by preparing the students psychologically. Later, we can provide them with the linguistic knowledge of English. So, I think psychology comes first after receiving knowledge and practicing it. I prefer the communicative approach because I think it is the only way that helps learners to speak real English or any foreign languages.

With regard to TK and TPK, the interview indicated that teachers passionately believed in integrating technology; they showed their enthusiasm and provided typical examples of its use in teaching. A large number of them stated that employing technology is very helpful as it makes classroom environment more enjoyable and motivates learners. This is in line with the findings of Lam (2000) who noted that the participants in his study considered technology from a ‘utilitarian perspective’. However, it is important to note that earlier in the interview teachers were questioned their beliefs about PK and CK, and there was hardly any teacher to talk about technology integration and its importance as a part of their career. This seems to imply that exploiting technology may not play a key role in their pedagogy. The finding that TK and TPK obtained the lowest mean in the quantitate phase, provides some evidence that the teachers experienced difficulties with adapting and employing technology in their educational environment. This concurs with Vereshchahina et al. (2018), showing that university teachers in their study had higher CK and PK than TK and TPK.

As shown in the quantitative analysis, teachers had a higher level of PK and CK; a lower mean was obtained for TK and technology-related knowledge domains (i.e., TK, TPK, TCK, and TPACK). This finding suggests that higher level of CK and PK is associated with the tendency to deliver a lecture and does not necessarily reflect teachers’ reliance on technology. Apparently, although teachers’ prevailing belief was to employ technology in the classroom, contextual barriers like the absence of high-speed internet and lack of modern equipment may prevent them from fulfilling this purpose. This finding is in line with Chen and Jangs’ (2014, 2019) study who found that CK, PK, PCK, had a higher mean score than technology-related components.

Regarding the use of current technologies, teachers noted that they use PowerPoint, projector, recorder, and YouTube in their classes; a large number of participants mentioned the use of social media channels like Messenger, Viber, and Telegram as the most common platforms for communication between teachers and learners, and learners themselves. Although some of these applications are mainly created for social activities and may not properly fulfill educational goals. Overreliance on social media channels rather than widespread e-learning platforms indicates that teachers have limited opportunities to properly grasp present-day educational technologies. Being incompetent in incorporating modern technology highlights the fact that the effectiveness of technology integration in classroom is largely determined by firm adherence to each constituent of TPACK (Adipat, 2021). As for the purpose of employing technology, the teachers stated that employing technology makes learning fun, saves time, and motivates their learners. This concurs with the Vereshchahina et al.’s (2018) and Singh and Kasims’ (2019) study who noted that employing technology saves teachers’ time and promotes learners’ motivation.

With regard to the second question, experienced teachers obtained significantly higher score for CK, PK, TK, and PCK. They also provided better conceptualizations of CK in the interviews. This could be related to the fact that additional years of teaching had offered them more time and opportunity to deal with a wider range of materials for language learning which consequently led them to assign a clearer meaning. This finding is in harmony with Jang and Chang (2016) who found that experienced teachers rated their subject matter knowledge and instructional strategies (i.e., CK and PK) higher than novice teachers.

Although the quantitative phase showed that experienced teachers had significantly higher score for TK, the interviews revealed that novice teachers felt more comfortable with integrating technology into their classes. For example, one of the teachers stated that “definitely, there is a strong need to blend technology in teaching because it facilitates the process of teaching and learning and helps students to learn faster and better.” Another novice teacher commented:

Using Technology in English classes gives authenticity to the learning process. The teachers can show footage of native speakers during real communication so the students will be exposed to the real English used by real people; they are exposed to real communication and pronunciation.

Similarly, the majority of experienced teachers believed that technology assists learning; nevertheless, some of them stated that they employed technology because their learners were using it rather than perceiving it as an essential need. It can be inferred that experienced teachers’ were playing a less active role in integrating technology while novice teachers showed more enthusiasm, provided more concrete examples, and yielded more profound reasons for using technology. Perhaps it is because novice teachers are generally younger than experienced teachers and are more inclined to use technology (Bennett et al., 2008); accordingly, they are more eager to utilize technology in their teaching.

As for TPK, it should be noted that the experienced teachers used some terms like ‘give guidelines,’ ‘recommend,’ ‘encourage,’ ‘teach,’ ‘share information,’ and ‘discuss’. The novice teachers, on the contrary, more frequently used ‘encourage,’ ‘watch movies,’ ‘read e-books,’ ‘instruct,’ ‘introduce,’ ‘prepare,’ and ‘share.’ Generally speaking, it sounds like the novice teachers attached a more active role to incorporating technology in their teaching than the experienced teachers. Additionally, they benefitted from technology for a wider range of functions and activities, which could be because of their mastery over technological devices and programs. Apparently, because novice teachers are more immersed with technology and nearly the ‘net generation’ (Bennett et al., 2008), they tend to have better mastery over technology-related knowledge domains. This concurs with the findings of Nazari et al. (2019) and Singh and Kasims’ (2019) study who found that novice teachers integrate technology to make learning more meaningful.

Concerning educational degrees of the participants, the quantitative phase indicated that teachers who studied doctorate have significantly higher mean score than BA teachers. The results also indicated that the teachers were significantly different in terms of TK in which Ph.D. and MA holders had a higher level of TK than the BA holders. This might be because Ph.D. and MA participants had passed some courses on integrating technology in their classes. They tend to teach university students or adult learners which mostly seek technology to present the materials and to make contact with the students. On the other hand, teachers at the BA level are mostly teach school children or younger learners; they may think that it is not as mush necessary as it is for more grown learners. Even if BA holders have used technology more intensively in teaching during the COVID pandemic, participants with MA and Ph.D. degrees have a wealth of experience in doing so. As a result, more experience has led them to more competency, especially in mastering educational technology. This concurs with Luik et al.’s (2018) results who found that MA level participants had generally higher perception of TPACK than participants at the BA level.

## Conclusion and implications

The present study investigated teachers’ beliefs, understandings, and uses of TPACK and its components. For this purpose, teachers’ general assumptions, experience, and academic degrees were taken into account. The findings indicated that Iraqi Kurdish teachers focused on various elements of TPACK for teaching subject contents and material presentations. However, contextual problems and lack of technology education have limited their use of technology. In the case of teaching experience, a great level of diversity in teachers’ beliefs was found. Experienced teachers had higher level of TPACK, especially in areas where technology were not involved. Regarding technology-related knowledge domains, novice teachers expressed much greater confidence and readiness to integrate technology in teaching and learning activities. Much like experience, education degrees had also created impact on understanding TPACK; Ph.D. and MA participants had greater preference for integrating technology in teaching than BA level participants.

In the light of the findings, it is recommended that institutions and universities offer intensive courses for teachers, especially experienced teachers, and introduce them to current digital technologies and their uses. As novice teachers lack pedagogical strategies and experienced teachers need more technological support, collaborations between the both is highly recommended. To promote teachers’ digital literacy in content representation and knowledge transition, it is essential to provide them with feedback regarding the integration of technology in practice.

## Data availability statement

The raw data supporting the conclusions of this article will be made available by the authors, without undue reservation.

## Ethics statement

Ethical review and approval was not required for the study on human participants in accordance with the local legislation and institutional requirements. The patients/participants provided their written informed consent to participate in this study.

## Author contributions

SA collected the data, wrote the paper, and performed the analysis. BM conceived and designed the analysis, wrote the paper, and performed the analysis. All authors contributed to the article and approved the submitted version.

## Conflict of interest

The authors declare that the research was conducted in the absence of any commercial or financial relationships that could be construed as a potential conflict of interest.

## Publisher’s note

All claims expressed in this article are solely those of the authors and do not necessarily represent those of their affiliated organizations, or those of the publisher, the editors and the reviewers. Any product that may be evaluated in this article, or claim that may be made by its manufacturer, is not guaranteed or endorsed by the publisher.

## References

[ref1] Adipat S. (2021). Developing technological pedagogical content knowledge (TPACK) through technology-enhanced content and language-integrated learning (T-CLIL) instruction. Educ. Inf. Technol. 26, 6461–6477. doi: 10.1007/s10639-021-10648-3, PMID: 34220281 PMC8234760

[ref2] Anderson S. E. Putman R. S. (2020). Special education teachers, experience, confidence, beliefs, and knowledge about integrating technology. J. Spec. Educ. Technol. 35, 37–50. doi: 10.1177/0162643419836409

[ref3] Aniq L. N. Drajati N. A. (2019). Investigating EFL teachers, perceptions on their TPACK development: how EFL teachers view seven domains on TPACK framework. Leksika: Jurnal Bahasa, Sastra Dan Pengajarannya 13, 95–101. doi: 10.30595/lks.v13i2.5649

[ref4] Archambault L. M. Barnett J. H. (2010). Revisiting technological pedagogical content knowledge: exploring the TPACK framework. Comput. Educ. 55, 1656–1662. doi: 10.1016/je.compedu.2010.07.009

[ref6] Baser D. Kopcha T. J. Ozden M. Y. (2016). Developing a technological pedagogical content knowledge (TPACK) assessment for preservice teachers learning to teach English as a foreign language. Comput. Assist. Lang. Learn. 29, 749–764. doi: 10.1080/09588221.2015.1047456

[ref7] Bennett S. Maton K. Kervin L. (2008). The “digital natives” debate: a critical review of the evidence. Br. J. Educ. Technol. 39, 775–786. doi: 10.1111/j.1467-8535.2007.00793.x

[ref8] Borg S. (2003). Teacher cognition in language teaching: a review of research on what language teachers think, know, believe, and do. Lang. Teach. 36, 81–109. doi: 10.1017/S0261444803001903

[ref9] Burns A. Freeman D. Edwards E. (2015). Theorizing and studying the language-teaching mind: mapping research on language teacher cognition. Mod. Lang. J. 99, 585–601. doi: 10.1111/modl.12245

[ref10] Cahapay M. B. (2020). Rethinking education in the new normal post-COVID-19 era: a curriculum studies perspective. Aqua 4, 2–5. doi: 10.29333/aquademia/8315

[ref11] Chai C. S. Koh J. H. L. Tsai C. C. (2016). “A review of the quantitative measures of technological pedagogical content knowledge (TPACK)” in Handbook of Technological Pedagogical Content Knowledge (TPACK) for Educators. eds. Herring M. C. Koehler M. J. Routledge P. M. (New York: London), 87–106.

[ref12] Chai C. S. Hwee Ling Koh J. Teo Y. H. (2019). Enhancing and modeling teachers, design beliefs and efficacy of technological pedagogical content knowledge for 21st century quality learning. J. Educ. Comput. Res. 57, 360–384. doi: 10.1177/0735633117752453

[ref13] Chauhan S. (2017). A meta-analysis of the impact of technology on learning effectiveness of elementary students. Comput. Educ. 105, 14–30. doi: 10.1016/j.compedu.2016.11.005

[ref14] Chen Y. H. Jang S. J. (2014). Interrelationship between stages of concern and technological, pedagogical, and content knowledge: a study on Taiwanese senior high school in-service teachers. Comput. Hum. Behav. 32, 79–91. doi: 10.1016/j.chb.2013.11.011

[ref15] Chen Y. H. Jang S. J. (2019). Exploring the relationship between self-regulation and TPACK of Taiwanese secondary in-service teachers. J. Educ. Comput. Res. 57, 978–1002. doi: 10.1177/0735633118769442

[ref16] Dong Y. Chai C. S. Sang G. Y. Koh J. H. L. Tsai C. C. (2015). Exploring the profiles and interplays of pre-service and in-service teachers' technological pedagogical content knowledge (TPACK) in China. J. Educ. Technol. Soc. 18, 158–169. doi: 10.25273/pe.v11i1.7882

[ref17] Ellis R. (2012). Language Teaching Research and Language Pedagogy. Hoboken, NJ: John Wiley & Sons. doi: 10.1002/9781118271643

[ref18] Emerson R. W. (2015). Convenience sampling, random sampling, and snowball sampling: how does sampling affect the validity of research? J. Vis. Impair. Blind. 109, 164–168. doi: 10.1177/0145482X1510900215

[ref19] Ertmer P. A. Ottenbreit-Leftwich A. T. Sadik O. Sendurur E. Sendurur P. (2012). Teacher beliefs and technology integration practices: a critical relationship. Comput. Educ. 59, 423–435. doi: 10.1016/j.compedu.2012.02.001

[ref20] Gatbonton E. (2008). Looking beyond teachers, classroom behaviour: novice and experienced ESL teachers' pedagogical knowledge. Lang. Teach. Res. 12, 161–182. doi: 10.1177/1362168807086286

[ref21] Gil-Flores J. Rodríguez-Santero J. Torres-Gordillo J.-J. (2017). Factors that explain the use of ICT in secondary education classrooms: the role of teacher characteristics and school infrastructure. Comput. Hum. Behav. 68, 441–449. doi: 10.1016/j.chb.2016.11.057

[ref22] Graham C. R. (2011). Theoretical considerations for understanding technological pedagogical content knowledge (TPACK). Comput. Educ. 57, 1953–1960. doi: 10.1016/j.compedu.2011.04.010

[ref23] Gunbas N. Gozukucuk M. (2020). Views of elementary school children,s parents about distance education during the Covid-19 pandemic. Sakarya Univ. J. Educ. 10, 686–716. doi: 10.19126/suje.789705

[ref24] Habibi A. Yusop F. D. Razak R. A. (2019). The role of TPACK in affecting preservice language teachers' ICT integration during teaching practices: Indonesian context. Educ. Inf. Technol. 25, 1929–1949. doi: 10.1007/s10639-019-10040-2

[ref25] Harris J. Phillips M. Koehler M. Rosenberg J. (2017). TPCK/TPACK research and development: past, present, and future directions. Australas. J. Educ. Technol. 33, i–viii. doi: 10.14742/ajet.3907

[ref26] Heitink M. Voogt J. Fisser P. Verplanken L. van Braak J. (2017). Eliciting teachers’ technological pedagogical knowledge. Australas. J. Educ. Technol. 33, 96–109. doi: 10.14742/ajet.3505

[ref27] Hsu L. (2016). Examining EFL teachers’ technological pedagogical content knowledge and the adoption of mobile-assisted language learning: a partial least square approach. Comput. Assist. Lang. Learn. 29, 1287–1297. doi: 10.1080/09588221.2016.1278024

[ref28] Hsu C. Y. Liang J. C. Chuang T. Y. Chai C. S. Tsai C. C. (2021). Probing in-service elementary school teachers’ perceptions of TPACK for games, attitudes towards games, and actual teaching usage: a study of their structural models and teaching experiences. Educ. Stud. 47, 734–750. doi: 10.1080/03055698.2020.1729099

[ref29] Jang S. J. Chang Y. (2016). Exploring the technical pedagogical and content knowledge (TPACK) of Taiwanese university physics instructors. Australas. J. Educ. Technol. 32, 107–122. doi: 10.14742/ajet.2289

[ref30] Jimoyiannis A. Komis V. (2007). Examining teachers' beliefs about ICT in education: implications of a teacher preparation programme. Teach. Dev. 11, 149–173. doi: 10.1080/13664530701414779

[ref31] Jin L. Xu Y. Deifell E. Angus K. (2021). Emergency remote language teaching and U.S.-based college-level world language Educators' intention to adopt online teaching in Postpandemic times. Mod. Lang. J. 105, 412–434. doi: 10.1111/modl.12712

[ref32] Koehler M. Mishra P. (2009). What is technological pedagogical content knowledge (TPACK)? Contemp. Issues Technol. Teach. Educ. 9, 60–70. doi: 10.1177/002205741319300303

[ref33] Koehler M. J. Mishra P. Yahya K. (2007). Tracing the development of teacher knowledge in a design seminar: integrating content, pedagogy and technology. Comput. Educ. 49, 740–762. doi: 10.1016/j.compedu.2005.11.012

[ref34] Koh J. H. L. Chai C. S. (2016). Seven design frames that teachers use when considering technological pedagogical content knowledge (TPACK). Comput. Educ. 102, 244–257. doi: 10.1016/j.compedu.2016.09.003

[ref35] Koh J. H. L. Chai C. S. Tsai C. C. (2010). Examining technological pedagogical content knowledge of Singapore preservice teachers with a large-scale survey. J. Comput. Assist. Learn. 26, 563–573. doi: 10.1111/j.1365-2729.2010.00372.x

[ref001] Lam Y. (2000). Technophilia vs Technophobia: A preliminary look at why second-language teachers do not use technology in their classrooms. Cana. Mod. Lang. Rev. 56, 390–420. doi: 10.3138/cmlr.56.3.389

[ref36] Lee M. H. Tsai C. C. (2010). Exploring teachers' perceived self efficacy and technological pedagogical content knowledge with respect to educational use of the world wide web. Instr. Sci. 38, 1–21. doi: 10.1007/s11251-008-9075-4

[ref37] Luik P. Taimalu M. Suviste R. (2018). Perceptions of technological, pedagogical and content knowledge (TPACK) among preservice teachers in Estonia. Educ. Inf. Technol. 23, 741–755. doi: 10.1007/s10639-017-9633-y

[ref38] Mishra P. Koehler M. J. (2006). Technological pedagogical content knowledge: a framework for teacher knowledge. Teach. Coll. Rec. 108, 1017–1054. doi: 10.1111/j.1467-9620.2006.00684.x

[ref39] Mishra P. Koehler M. J. (2008). “Introducing technological pedagogical content knowledge” in In Annual Meeting of the American Educational Research Association. eds. Mishra P. Hall E. (College of Education: Michigan State University), 1–16.

[ref40] Moser K. Wei T. (2021). COVID-19 and the pre-existing language teacher supply crisis. Lang. Teach. Res. 97, 1–15. doi: 10.1177/13621688211040297

[ref41] Moser K. M. Wei T. Brenner D. (2021). Remote teaching during COVID-19: implications from a national survey of language educators. System 97:102431. doi: 10.1016/j.system.2020.102431

[ref42] Nazari N. Nafissi Z. Estaji M. Marandi S. S. (2019). Evaluating novice and experienced EFL teachers’ perceived TPACK for their professional development. Cogent Educ. 6:1632010. doi: 10.1080/2331186X.2019.1632010

[ref43] Paratore J. R. O’Brien L. M. Jimenez L. Salinas A. Ly C. (2016). Engaging preservice teachers in integrated study and use of educational media and technology in teaching reading. Teach. Teach. Educ. 59, 247–260. doi: 10.1016/j.tate.2016.06.003

[ref44] Ponce Gea A. I. Rico Gómez M. L. Sola Reche J. M. García Vidal M. (2021). Teacher training about information and communication technologies: a diachronic perspective. Revista ESPACIOS 42, 189–200. doi: 10.48082/espacios-a21v42n01p16

[ref45] Prensky M. (2001). Digital natives, digital immigrants: part 1. Horizon 9, 1–6. doi: 10.1108/10748120110424816

[ref46] Raygan A. Moradkhani S. (2020). Factors influencing technology integration in an EFL context: investigating EFL teachers’ attitudes, TPACK level, and educational climate. Comput. Assist. Lang. Learn. 33, 1–22. doi: 10.1080/09588221.2020.1839106

[ref47] Santos J. M. Castro R. D. (2021). Technological pedagogical content knowledge (TPACK) in action: application of learning in the classroom by preservice teachers (PST). Soc. Sci. Human. Open 3:100110. doi: 10.1016/j.ssaho.2021.100110

[ref48] Schmidt D. A. Baran E. Thompson A. D. Mishra P. Koehler M. J. Shin T. S. (2009). Technological pedagogical content knowledge (TPACK) the development and validation of an assessment instrument for preservice teachers. J. Res. Technol. Educ. 42, 123–149. doi: 10.1080/15391523.2009.10782544

[ref49] Shulman L. S. (1986). Those who understand: knowledge growth in teaching. Educ. Res. 15, 4–14. doi: 10.3102/0013189X015002004

[ref50] Singh C. K. S. Kasim Z. M. (2019). Pre-service teachers’ mastery of technological pedagogical content knowledge for teaching English language. Univ. J. Educ. Res. 7, 24–29. doi: 10.13189/ujer.2019.071705

[ref51] Skott J. (2014). “The promises, problems and prospects of research on teachers’ beliefs,” in International Handbook of Research on Teachers’ Beliefs. eds. Fives H. Gill M. G. (London, United Kingdom: Routledge), 13–30.

[ref52] Szeto E. Cheng A. Y. N. (2017). Pedagogies across subjects: what are preservice teachers’ TPACK patterns of integrating technology in practice? J. Educ. Comput. Res. 55, 346–373. doi: 10.1177/0735633116667370

[ref53] Teo T. Huang F. Hoi C. K. W. (2018). Explicating the influences that explain intention to use technology among English teachers in China. Interact. Learn. Environ. 26, 460–475. doi: 10.1080/10494820.2017.1341940

[ref54] Tondeur J. van Braak J. Ertmer P. A. Ottenbreit-Leftwich A. (2017). Understanding the relationship between teachers' pedagogical beliefs and technology use in education: a systematic review of qualitative evidence. Educ. Technol. Res. Dev. 65, 555–575. doi: 10.1007/s11423-016-9481-2

[ref55] Tria J. Z. (2020). The COVID-19 pandemic through the lens of education in the Philippines: the new normal. Int. J. Pedagog. Dev. Lifelong Learning 1, 2–4. doi: 10.30935/ijpdll/8311

[ref56] Tseng J. J. Chai C. S. Tan L. Park M. (2020). A critical review of research on technological pedagogical and content knowledge (TPACK) in language teaching. Comput. Assist. Lang. Learn. 33, 1–24. doi: 10.1080/09588221.2020.1868531

[ref57] Vereshchahina T. Liashchenko O. Babiy S. (2018). English language teachers’ perceptions of hybrid learning at university level. Adv. Educ. 5, 88–97. doi: 10.20535/2410-8286.148368

[ref58] Voithofer R. Nelson M. J. (2021). Teacher educator technology integration preparation practices around TPACK in the United States. J. Teach. Educ. 72, 314–328. doi: 10.1177/0022487120949842

[ref59] Voogt J. Fisser P. Pareja Roblin N. Tondeur J. van Braak J. (2013). Technological pedagogical content knowledge–a review of the literature. J. Comput. Assist. Learn. 29, 109–121. doi: 10.1111/j.1365-2729.2012.00487.x

[ref60] West A. Swanson J. Lipscomb L. (2017). “Ch. 11 Scaffolding” in Instructional Methods, Strategies and Technologies to Meet the Needs of All Learners (Concord, NH: Granite State College)

[ref61] Willermark S. (2018). Technological pedagogical and content knowledge: a review of empirical studies published from 2011 to 2016. J. Educ. Comput. Res. 56, 315–343. doi: 10.1177/0735633117713114

[ref62] Wu Y. T. (2013). Research trends in technological pedagogical content knowledge (TPACK) research: a review of empirical studies published in selected journals from 2002 to 2011. Br. J. Educ. Technol. 44, E73–E76. doi: 10.1111/j.1467-8535.2012.01349.x

[ref63] Yenkimaleki M. van Heuven V. J. (2019). The relative contribution of computer assisted prosody training vs. instructor based prosody teaching in developing speaking skills by interpreter trainees: an experimental study. Speech Comm. 107, 48–57. doi: 10.1016/j.specom.2019.01.006

